# A Durable Remission Following Pseudo‐Progression in Tirabrutinib Treatment for Relapsed Primary Central Nervous System Lymphoma: A Case Study

**DOI:** 10.1155/crh/6823465

**Published:** 2025-12-05

**Authors:** Sayoko Okawara, Satoru Ide, Hiroaki Morimoto, Junichi Tsukada

**Affiliations:** ^1^ Department of Hematology, University of Occupational and Environmental Health, Kitakyushu City, Japan, uoeh-u.ac.jp; ^2^ Department of Radiology, University of Occupational and Environmental Health, Kitakyushu City, Japan, uoeh-u.ac.jp

**Keywords:** BTKi, lymphoma, PCNSL, pseudo-progression, refractory, tirabrutinib

## Abstract

Tirabrutinib (TIR) is a second‐generation, Bruton’s tyrosine kinase inhibitor (BTKi) recently developed for the treatment of relapsed and refractory primary central nervous system lymphoma (PCNSL). However, little data are available regarding potential immunomodulatory effects of TIR on PCNSL due to its rarity and aggressive tumor behavior. Here, we report the first case of pseudo‐progression (PSP) in a PCNSL patient treated with TIR. A 79‐year‐old woman had relapsed PCNSL with multiple tumor lesions in the lateral ventricles. A temporary tumor regression was observed following TIR administration. However, 7 months later, brain tumors regrew in the left lateral ventricle and in the choroid plexus of the right lateral ventricle, suggesting TIR‐resistant disease progression. Despite the enlarged tumors, the patient remained asymptomatic, and re‐remission was achieved by continuation of TIR monotherapy. Moreover, a durable remission for approximately 2 years was obtained without any additional therapy. This case shows that TIR can induce immunomodulatory reaction including PSP even in PCNSL, suggesting the importance of differential diagnosis of true disease progression and immune‐mediated PSP based on careful clinical and radiological monitoring to avoid premature discontinuation of effective treatment.

## 1. Introduction

Tirabrutinib (TIR) is a second‐generation, highly selective Bruton’s tyrosine kinase inhibitor (BTKi), which is approved as a novel treatment for relapsed and refractory (r/r) primary malignant lymphoma of the central nervous system (PCNSL) in Japan, South Korea, and Taiwan [1, 2]. The PROSPECT study, an open‐label Phase II trial for evaluating the safety and efficacy of TIR for patients with newly diagnosed or r/r PCNSL in the United States (NCT04947319) is already in progress. This agent has been reported to have an overall response rate of 63.6%, including 36.4% complete response (CR) [1, 2].

On the other hand, immunomodulatory effects of ibrutinib, a first generation BTKi has been recently reported in the treatment for chronic lymphocytic leukemia (CLL)/small lymphocytic lymphoma (SLL) [3, 4], marginal zone lymphoma (MZL) [5], and follicular lymphoma (FL) [6]. However, little is known about the potential immune‐related effects of TIR due to the rarity and aggressive tumor behavior of PCNSL. Here, we present a case of a patient with r/r PCNSL, who developed pseudo‐progression (PSP) following TIR administration. After PSP, a durable remission was obtained by continuing administration of TIR.

## 2. Case Presentation

A 79‐year‐old woman presented with intermittent headache and short‐term memory impairment. She had no significant medical history. No superficial lymph nodes were enlarged. Blood counts showed white blood cells 4.3 × 10^9^/L (neutrophils 76%, lymphocytes 20%, and monocytes 4%), hemoglobin 12.5 g/dL, and platelets 123 × 10^9^/L. Serum lactate dehydrogenase (LDH) and soluble interleukin‐2 receptor (sIL‐2R) were normal, and HIV antibodies were negative. Contrast‐enhanced magnetic resonance imaging (MRI) of the head revealed multiple brain tumors with diffuse contrast enhancement in the walls and the choroid plexus of bilateral lateral ventricles (Figure 1(a)), while no tumor lesions were found in whole‐body contrast‐enhanced computed tomography, bone marrow examination, and ophthalmologic examination. Abnormal large lymphoid cell invasion was observed in the stereotactic brain tumor biopsy (Figure 1(b)). The lymphoid cells were positive for CD20, BCL2, BCL6, and MUM1 and negative for CD3 and CD10. The Ki‐67‐proliferative fraction (MIB‐1 index) was 80% (Figure 1(b)). The patient was diagnosed as primary diffuse large B‐cell lymphoma non–GC‐type of the CNS. Temporary tumor reduction was obtained with R‐MPV therapy (rituximab, methotrexate (MTX), procarbazine, and vincristine) with intrathecal MTX, cytarabine and prednisolone. However, after 5 cycles of R‐MPV, her headache was worsened, and MRI revealed enlarged tumors in the bilateral anterior horns and the right triangular to posterior horns of the lateral ventricles (Figure 2(a)).

Figure 1Primary central nervous system lymphoma at the initial diagnosis. (a) Magnetic resonance imaging of the brain at the initial diagnosis. Gadolinium‐enhanced T1‐weighted images showed a high‐intensity multiple lesions in the ventricles (red circles and red arrows). (b) Pathological findings of the tumor: original magnification X200. Larger‐sized atypical lymphoid cells diffusely invaded the tissue. Immunohistochemically, the atypical cells were positively reactive to CD20, Bcl‐2, Bcl‐6, and MUM1, whereas they do not express CD3 or CD10. MIB‐1 labeling index was approximately 80%. CD3‐positive T‐cells were admixed.

**Figure:**
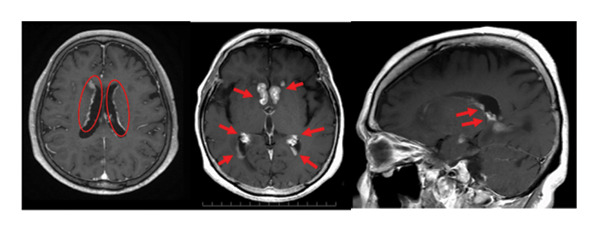
(a)

**Figure:**
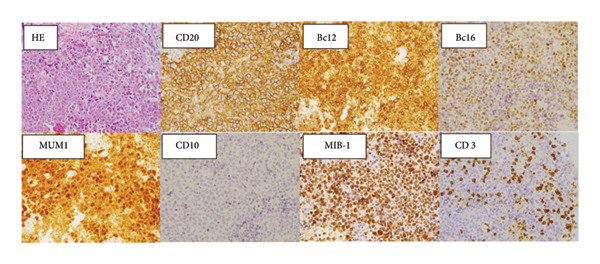
(b)

Figure 2Gadolinium‐enhanced T1‐weighted magnetic resonance imaging (MRI) of the brain during tirabrutinib (TIR) therapy. (a) MRI before TIR treatment showed tumor enlargement in the bilateral anterior horns and the right triangular to posterior horns of the lateral ventricles (red arrows). (b) 3 weeks after the initiation of TIR, MRI showed improved lesions, although a small residual lesion was detected in the anterior horn of the right lateral ventricle (a red arrow). (c) 7 months after the TIR initiation, MRI showed the disappearance of the lesion in the anterior horn of the right lateral ventricle, but tumor progression was observed in the wall of the left lateral ventricle and in the choroid plexus of the right ventricle (red arrows). (d) 17 months after the TIR initiation, all the lesions disappeared.

**Figure:**
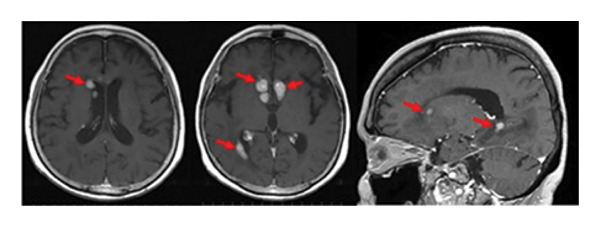
(a)

**Figure:**
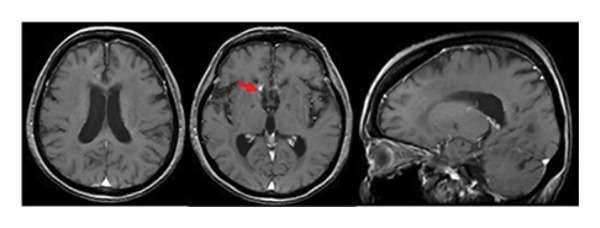
(b)

**Figure:**
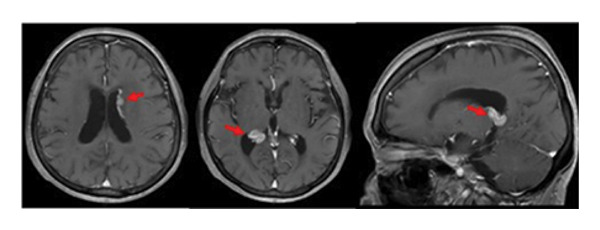
(c)

**Figure:**
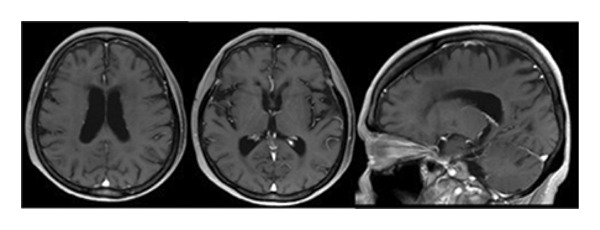
(d)

Oral administration of TIR at a dose of 480 mg/day was initiated as a salvage chemotherapy, and then the dose of TIR was reduced to 320 mg/day due to neutropenia Grade 3 and pneumonia. Three weeks after the initiation of TIR, although a small residual lesion was observed in the anterior horn of the right lateral ventricle, a significant tumor regression was obtained (Figure 2(b)). However, 7 months after the TIR initiation, tumor progression was observed in the wall of the left lateral ventricle and in the choroid plexus of the right ventricle, in contrast to tumor regression in the lesion of the anterior horn of the right lateral ventricle (Figure 2(c)). The patient had a good performance status and did not show any significant symptoms such as headache or nausea. Serum LDH (209 U/mL) and sIL‐2R (352 U/mL) levels were also normal. Therefore, she and her family favored continuation of TIR and refused rebiopsy. Five months later (12 months after the TIR initiation), continuation of TIR resulted in almost disappearance of the tumors in all the lesions. As shown in Figure 2(d), 17 months after the TIR initiation, CR was confirmed.

During the 10‐month period, no additional treatments including steroids have been administered. At the time of the initial diagnosis of PCNSL, the lesions in the anterior horn of the right and left lateral ventricle (Figure 3(a)) showed diffusion restriction with a mean apparent diffusion coefficient (ADC) value of 634–809 × 10^−6^ mm^2^/s (Figure 3(b)). Seven months after the initiation of TIR, new hyperintense lesions appeared in the right choroid plexus and along the wall of the left lateral ventricle (Figure 3(c) and 3(e)), with a slightly increased mean ADC value of 807–1191 × 10^−6^ mm^2^/s (Figure 3(d) and 3(f)). These measurements were obtained by manually placing polygonal regions of interest on the hyperintense areas seen on diffusion weighted imaging (DWI) (Figure 3(a)–3(e) and Supporting). Furthermore, the means of ADC values at the initial diagnosis (Figure 3(b) and Supporting) and at 7 months after the initiation of TIR (Figure 3(d) and 3(f)) were 795 × 10^−6^ mm^2^/s (standard deviation [SD], 124.7 × 10^−6^ mm^2^/s) and 976 × 10^−6^ mm^2^/s (SD, 160.3 × 10^−6^ mm^2^/s), respectively, demonstrating a significant difference between the two time points (two‐sided *t*‐test, *p* = 0.017). She has been in a good general condition without neurological symptoms during TIR therapy. In addition, preserved CD4+ and CD8+ T‐cells was found in flow cytometry analysis of peripheral blood lymphocytes. However, unfortunately, TIR was discontinued, since she was infected with COVID‐19. She died of tumor relapse 26 months after the TIR initiation.

Figure 3Diffusion‐weighted imaging (DWI) and apparent diffusion coefficient (ADC) maps at the initial diagnosis of primary central nervous system lymphoma and at 7 months after initiating tirabrutinib (TIR). (a) DWI at the initial diagnosis showed hyperintense lesions in the anterior horn of the right and the left lateral ventricle (red arrows). (b) Corresponding ADC map demonstrated restricted diffusion in the lesion, with mean ADC values of 634 × 10^−6^ mm^2^/s, 786 × 10^−6^ mm^2^/s and 809 × 10^−6^ mm^2^/s (white polygonal lines 1–3). (c, e) DWI obtained 7 months after the initiation of TIR showed new hyperintense lesions in the right choroid plexus and along the wall of the left lateral ventricle (red arrows). (d, f) The corresponding ADC map showed a slightly increased mean ADC values of 807 × 10^−6^ mm^2^/s, 1191 × 10^−6^ mm^2^/s, 928 × 10^−6^ mm^2^/s, and 979 × 10^−6^ mm^2^/s (white polygonal lines 1–4), suggesting reduced cellularity compared to the initial lesion.

**Figure:**
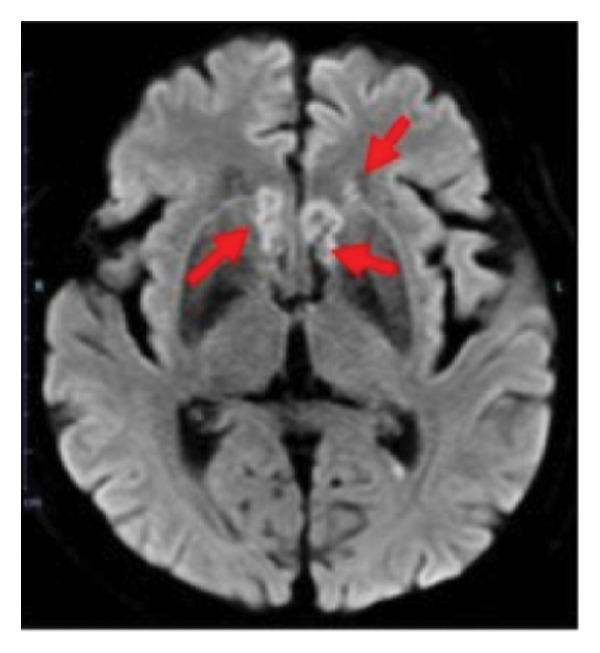
(a)

**Figure:**
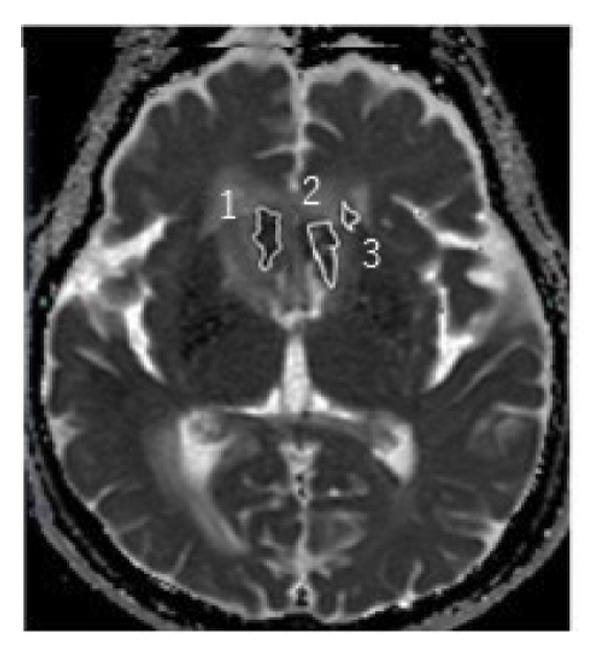
(b)

**Figure:**
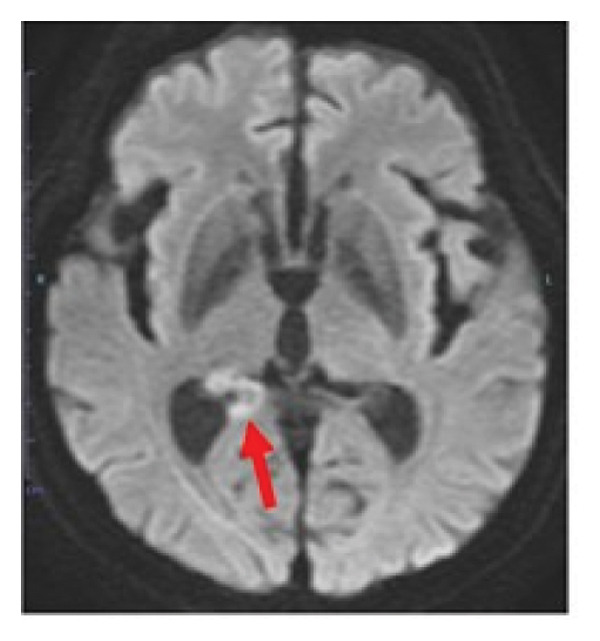
(c)

**Figure:**
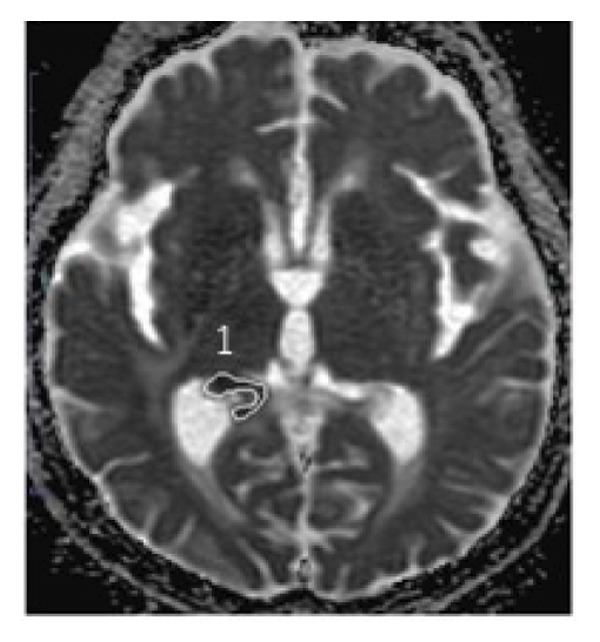
(d)

**Figure:**
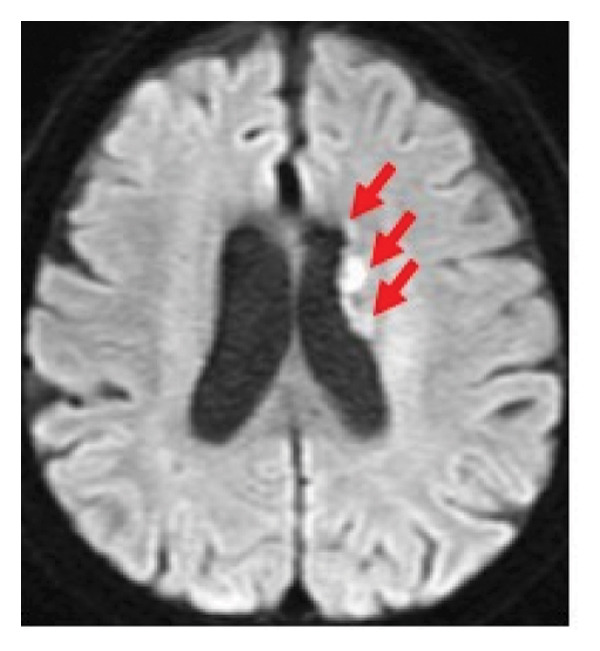
(e)

**Figure:**
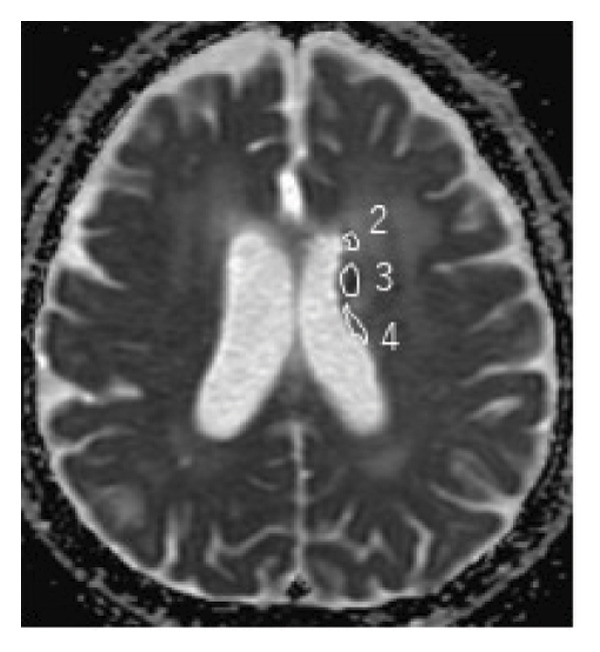
(f)

## 3. Discussion

PSP has been described primarily with immune checkpoint inhibitor therapy for solid tumors. In malignant lymphomas treated with immunomodulatory agents, Cheson et al. introduced the concept of ‘indeterminate response’ (IR) to reflect flare/PSP and proposed the lymphoma response to immunomodulatory therapy criteria (LYRIC) by modifying the Lugano criteria [7]. IR is defined by one or more of following: An increase in overall tumor burden of > 50% of up to six measurable lesions in the first 12 weeks of therapy, without clinical deterioration, IR (1); appearance of new lesions or growth of one or more existing lesions > 50% at any time during treatment occurring in the context of lack of overall progression of overall tumor burden, IR (2); an increase in fluorodeoxyglucose (FDG) uptake of one or more lesions without a concomitant increase in lesion size or number, IR (3). Our case was consistent with IR (2). The patient remained clinically asymptomatic, while growth of new lesions was observed in the ventricular walls and choroid plexus.

There are a few data regarding PSP/tumor flare in BTKi treatment for malignant lymphoma [5, 6, 8]. Typically, in CLL/SLL, peripheral blood lymphocytosis associated with a redistribution of lymphocytes from the tissues to the peripheral blood has been observed after the start of BTKi treatment [8–10]. On the other hand, the DAWN study reported that 7 patients with FL developed PSP during ibrutinib treatment. Among them, CR was achieved in four patients and partial response (PR) in three, and the responses were maintained in three for over 1 year and in two for more than 2 years [6]. PSP occurred in two of 63 ibrutinib‐treated patients with MZL at Weeks 9 and 13 [5].

Besides direct inhibition of B‐cell lymphoma, downregulation of regulatory T‐cells and repolarization of CD4‐positive T‐cells from Th‐2 to Th‐1 has been observed during ibrutinib treatment for FL [6] and CLL [3]. Moreover, the B‐cell receptor signaling is a critical signaling pathway for regulatory B‐cells (Breg), which suppress effector T‐cell functions. TIR [11] and ibrutinib [4] have been reported to suppress the differentiation and immunosuppressive functions of Breg and reduce the secretion of anti‐inflammatory cytokines, such as IL‐10. Reduced expression of PD‐1 on T‐cells and CLL cells [4] and inhibition of immunosuppressive functions of myeloid‐derived suppressor cells [12] have been also shown in ibrutinib treatment.

Consistent with our case, a Phase I study of pomalidomide (POM), a third‐generation immunomodulatory agent, and dexamethasone (Dex) for r/r PCNSL, and primary vitreoretinal lymphoma reported that one PCNSL patient developed PSP after Cycle 4 (4 months after the initiation of POM/Dex) [13]. Thus, the CNS may not be an exception to this phenomenon. Recent studies have demonstrated immune cell trafficking and lymphatic drainage of cerebrospinal fluid between the CNS and peripheral lymphatic tissues [14–16]. Our case also showed marked infiltration of T‐cells into the tumor at the initial diagnosis of PCNSL (Figure 1(b)).

Although histopathological confirmation was not feasible in our case, we carefully assessed the radiological and clinical features to support the diagnosis of PSP. In this regard, ADC values derived from DWI have been reported to inversely correlate with the histopathological assessment of tumor cell density in PCNSL [17]. In our case, the newly developed enhancing lesions in the right choroid plexus and along the wall of the left lateral ventricle showed diffusion restriction (Figure 3(c) and 3(e)), but its ADC value (Figure 3(d) and 3(f)) was slightly higher than that of the lesion observed at the time of the initial diagnosis of PCNSL (Figure 3(b) and Supporting). In glioma, it is well established that ADC values in PSP tend to be higher than those at initial diagnosis due to decreased cellular density [18, 19]. A similar interpretive approach may be reasonably applied to PCNSL, despite the lack of disease‐specific validation. Importantly, the lesion in our case regressed completely without any change in treatment, and the patient remained asymptomatic throughout the clinical course. These features support the interpretation of an immune‐related PSP.

In our case, PSP emerged 7 months post‐TIR initiation. Such delayed response was observed in the DAWN study of ibrutinib treatment for r/r FL, which reported post‐PSP remission at a median of 22 weeks after starting ibrutinib [6]. The Phase I/II study of TIR treatment for r/r PCNSL in Japan also revealed that some TIR responders achieved CR/CR undetermined (CRu) 6 months after the initiation of TIR [1, 2]. Thus, TIR may influence immune responses through improving the tumor microenvironment and restoring T‐cell functions, thereby reactivating endogenous anti‐PCNSL tumor activity as a delayed and durable response. In immunomodulatory therapy, these phenomena should be carefully considered to avoid premature cessation of effective drugs without achieving therapeutic benefit.

## Ethics Statement

This is a case report. The ethical committee of our institute has confirmed that ethical approval was not required.

## Consent

The patient verbally consented to deidentified material being shared in the form of this case report.

## Disclosure

All the authors critically read and made contributions to the final manuscript.

## Conflicts of Interest

The authors declare no conflicts of interest.

## Author Contributions

S.O. treated patients, gathered all figures, and described initial manuscripts. S.I. and H.M. analyzed the data and reviewed the manuscript. J.T. supervised treatment and reviewed the manuscript.

## Funding

This study received no funding from public, private, or not‐for‐profit sectors.

## Supporting Information

Supporting Information: Diffusion‐weighted imaging (DWI) and apparent diffusion coefficient (ADC) maps showing lesions where mean ADC values were measured at the initial diagnosis of primary central nervous system lymphoma, except for Figure 3(a). [B]4: 677 × 10^−6^ mm^2^/s, 5: 848 × 10^−6^ mm^2^/s, and 6: 557 × 10^−6^ mm^2^/s. [D]7: 587 × 10^−6^ mm^2^/s, 8: 761 × 10^−6^ mm^2^/s, 9: 830 × 10^−6^ mm^2^/s, 10: 779 × 10^−6^ mm^2^/s, and 11: 787 × 10^−6^ mm^2^/s. [F]12: 1016 × 10^−6^ mm^2^/s, 13: 716 × 10^−6^ mm^2^/s, 14: 822 × 10^−6^ mm^2^/s, 15: 831 × 10^−6^ mm^2^/s, and 16: 745 × 10^−6^ mm^2^/s. [H]17: 731 × 10^−6^ mm^2^/s, 18: 969 × 10^−6^ mm^2^/s, and 19: 963 × 10^−6^ mm^2^/s. [J]20: 843 × 10^−6^ mm^2^/s, and 21: 997 × 10^−6^ mm^2^/s.

## Supporting information


**Supporting Information** Additional supporting information can be found online in the Supporting Information section.

## Data Availability

The authors declare that data supporting the findings of this study are available within the article. Further inquiries can be directed to the corresponding author.
