# Supplementary figures and images for: A Durable Remission Following Pseudo‐Progression in Tirabrutinib Treatment for Relapsed Primary Central Nervous System Lymphoma: A Case Study
Source: Case Rep Hematol. 2025 Dec 5;2025:6823465. doi: 10.1155/crh/6823465 (PMC12721748; doi:10.1155/crh/6823465)

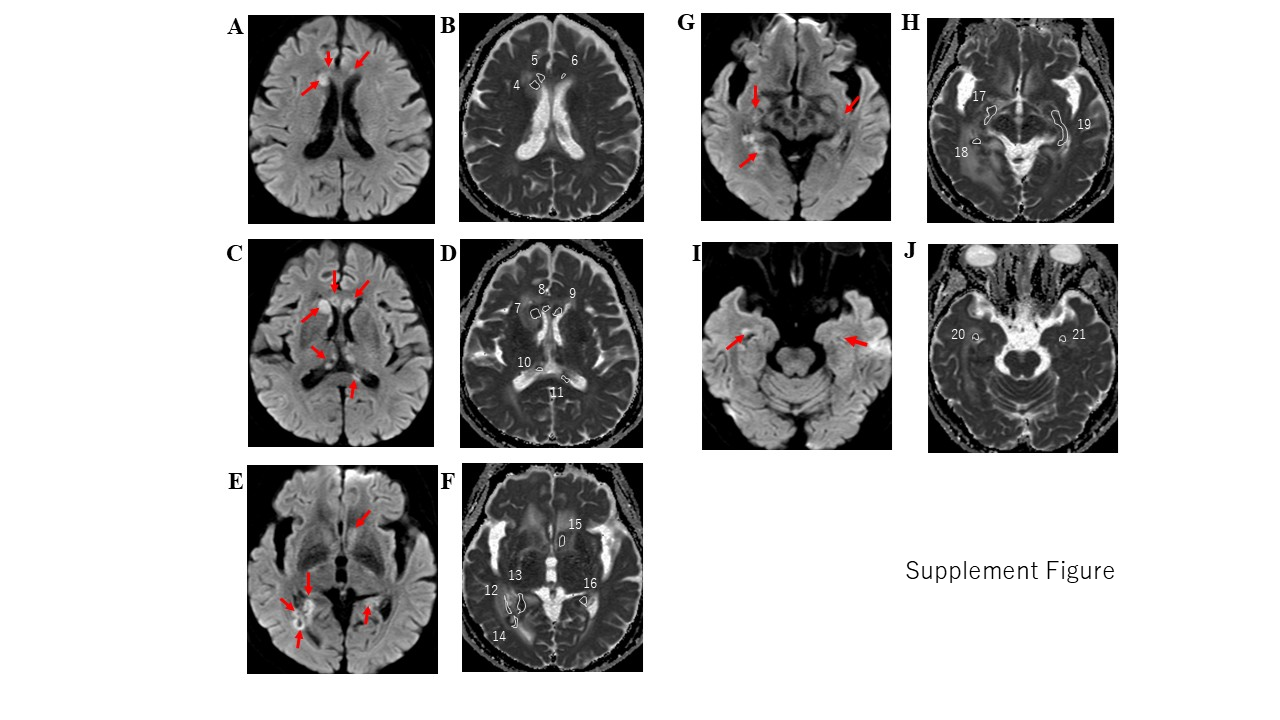

Supplement: Supplementary file 1 — Supporting Information Additional supporting information can be found online in the Supporting Information section. [file CRH-2025-6823465-s001.tif]
